# 4-(Di­methyl­amino)­benzohydrazide

**DOI:** 10.1107/S2414314620013103

**Published:** 2020-10-09

**Authors:** Steven P. Kelley, Valeri V. Mossine, Thomas P. Mawhinney

**Affiliations:** a Department of Chemistry, University of Missouri, Columbia, MO 65211, USA; b Department of Biochemistry, University of Missouri, Columbia, MO 65211, USA; University of Aberdeen, United Kingdom

**Keywords:** crystal structure, acyl hydrazide, hydrogen bonding, lattice energy, DFT calculations

## Abstract

The title compound, C_9_H_13_N_3_O, crystallizes in the monoclinic space group *C*2/*c* and all non-hydrogen atoms are within 0.1 Å of the mol­ecular mean plane. In the crystal, the hydrogen-bonding pattern results in [001] chains built up from fused *R*_2_^2^(6) and *R*_2_^2^(10) rings; the former consists of N—H⋯N bonds and the latter N—H⋯O bonds. Electrostatic and dispersion forces are major contributors to the lattice energy, which was estimated by DFT calculations to be −215.7 kJ mol^−1^.

## Abstract 2

The title mol­ecule is essentially flat; in the crystal the mol­ecules are linked by a system of hydrogen bonds formed by the hydrazido group and consisting of chains of fused rings.

## Graphical abstract

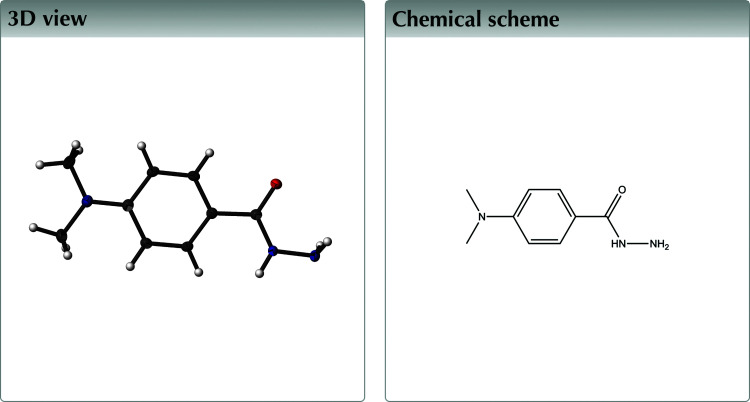

## Structure description

For decades, there has been an inter­est in aroyl hydrazides because of their numerous applications, for instance, as synthetic precursors to a large number of potential anti­microbial (Popiołek, 2017 ▸) or anti­cancer (Kumar & Narasimhan, 2013 ▸) drugs, in addition to their own anti-tubercular activities (Sah & Peoples, 1954 ▸). In our search for inhibitors of bacterial virulence factors (Mossine *et al.*, 2016 ▸, 2020 ▸), we turned our attention to the title compound, which can be viewed as a structural analogue of isoniazid (Andrade *et al.*, 2008 ▸) and a potential precursor for pharmacologically active, iron-binding hydrazide-hydrazones. We now report its crystal structure.

**Figure 1:**
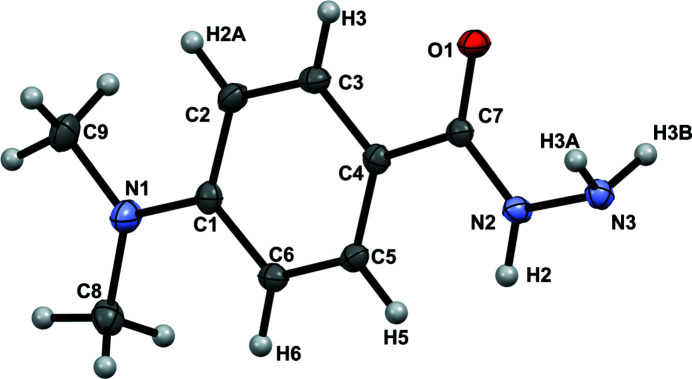
Atomic numbering and displacement ellipsoids at the 50% probability level for (**I**).

The title compound crystallizes in the monoclinic space group *C*2/*c*, with eight mol­ecules per unit cell. The asymmetric unit contains one mol­ecule of the hydrazide (**I**), as shown in Fig. 1 ▸. All bond lengths and angles are within their expected ranges. The mol­ecule is essentially flat, with the greatest deviation from the average mol­ecular plane, among the non-hydrogen atoms, found for atom N1 at 0.074 (1) Å. The aromatic ring plane is at 1.08 (4)° to the mol­ecular plane. The spatial arrangement of the hydrazido group, as defined by the torsion angle H2—N2—N3—H3*B* = 119.3 (15)°, corresponds to the lowest energy conformation that has been calculated for acyl hydrazides (Centore *et al.*, 2010 ▸).

**Figure 2:**
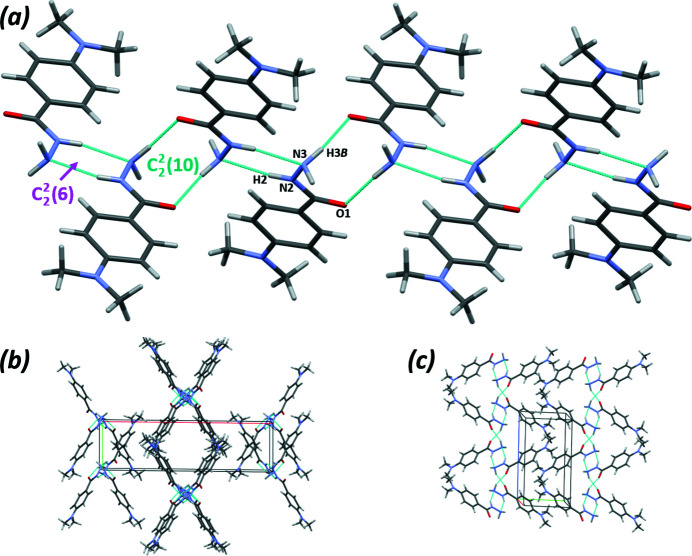
Mol­ecular packing and hydrogen bonding in (**I**). (*a*) Hydrogen-bonding motifs; (*b*) and (*c*) mol­ecular packing views down [001] and [100], respectively. Hydrogen bonds are shown as cyan dotted lines.

**Figure 3:**
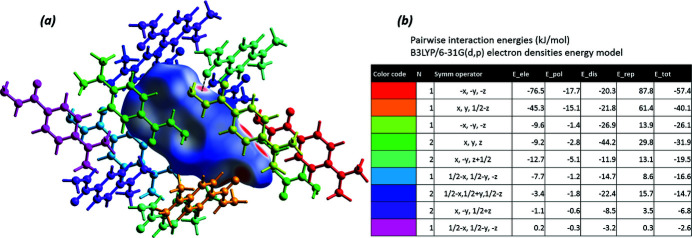
Inter­action energies in crystal structure of (**I**). (*a*) A view of inter­actions between a central mol­ecule, shown as its Hirshfeld surface, and 13 mol­ecules that share the inter­action surfaces with the central mol­ecule. Red areas on the Hirshfeld surface encode the closest inter­molecular contacts, which are hydrogen bonds involving the hydrazido groups, a short C—H⋯O type contact is shown as a dotted line; (*b*) Calculated energies (electrostatic, polarization, dispersion, repulsion, and total) of pairwise inter­actions between the central mol­ecule and those indicated by respective colours.

The conventional hydrogen bonding in the extended structure of (**I**) is limited to two inter­molecular heteroatom contacts (Table 1 ▸) involving the hydrazido groups only and is shown in Fig. 2 ▸. The hydrogen-bonding pattern includes infinite chains that propagate in the [001] direction and consist of fused 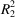 (10) and 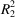 (6) rings (Fig. 2 ▸ *a*). The 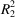 (10) motif is formed by pairs of mol­ecules linked by the N3—H3*B*⋯O1 hydrogen bonds related by twofold rotation symmetry, while the 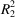 (6) motif is formed by centrosymmetric dimers of (**I**) linked by the N2—H2⋯N3 hydrogen bonds. In addition, one short inter­molecular contact, C6—H6⋯O1, which satisfies the distance and directionality conditions [C6⋯O1^iii^ = 3.4111 (13) Å, C6—H6⋯O1^iii^ = 172°; symmetry code: (iii) *x*, 1 − *y*, ½ + *z*], and which is shown in Fig. 3 ▸ as a dotted line, may also contribute to the stability of the mol­ecular packing in the crystal. The inter­molecular non-polar inter­actions are dominated by hydrogen–hydrogen contacts between the methyl groups; the shortest of these contacts, H8*C*⋯H9*B*, is about 0.1 Å less than the sum of the VdW radii. These inter­actions form a pattern of infinite chains, propagating in the [001] direction, in parallel to the hydrogen-bonded chains (Fig. 2 ▸ *b* and 2*c*). The crystal structure lacks any strong π–π stacking inter­actions. However, a short N3—H3*A*⋯*Cg*1 [H3*A*⋯*Cg*1^iv^ = 2.614 (15) Å; symmetry code: (iv) *x*, *y* − 1, *z*] contact is present.

**Figure 4:**
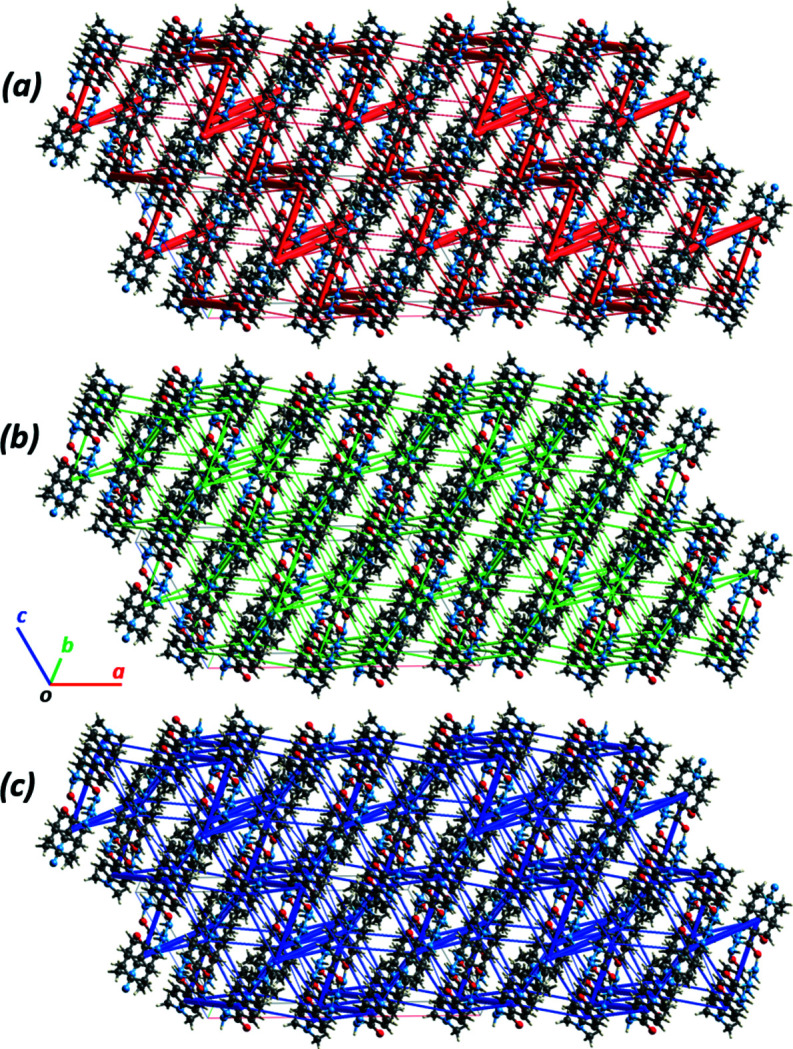
Energy frameworks for separate (*a*) electrostatic and (*b*) dispersion contributions to the (*c*) total pairwise inter­action energies in (**I**). The cylinders link mol­ecular centroids, and the cylinder thickness is proportional to the magnitude of the energies (see Fig. 3 ▸). For clarity, the cylinders corresponding to energies <5 kJ mol^−1^ are not shown. The directionality of the crystallographic axes is the same for all three diagrams.

To account for all inter­actions involved in the build-up of the crystal structure of (**I**) we have performed DFT calculations, at the B3LYP/6–31 G(d,p) theory level (Mackenzie *et al.*, 2017 ▸; Thomas *et al.*, 2018 ▸), of the electrostatic, dispersion, polarization, and repulsion energies. According to these calculations, the inter­actions between hydrogen-bonded pairs of mol­ecules contribute about 50% to the lattice energy, with the dispersion energy providing most of the attractive forces between non-hydrogen-bonded mol­ecules of (**I**) (*i.e. E*_ele_ = −9.2 kJ mol^−1^, *E*_dis_ = −44.2 kJ mol^−1^ for symmetry code = *x*, *y*, *z*). To estimate the lattice energy, all total energies of unique pairwise inter­actions between mol­ecules were summed up, thus yielding *E*_l_ (l = lattice) = −216 kJ mol^−1^ for the crystal of (**I**). The calculated contributions to the overall lattice energy (kJ mol^−1^) are as follows: *E*_ele_ = −165.3; *E*_pol_ = −46.0; *E*_dis_ = −173.9; *E*_rep_ = 234.1. The spatial distribution of the energetically most significant inter­actions is illustrated in Fig. 4 ▸, showing the inter­actions energy frameworks as cylinders penetrating the mol­ecular packing of (**I**). As expected, the most extensive inter­molecular inter­actions occur in the hydrogen-bonded chain direction parallel to [001].

## Synthesis and crystallization

A sample of commercial 4-di­methyl­amino­benzhydrazide was recrystallized from hot 95% ethanol solution, affording colorless needles.

## Refinement

Crystal data, data collection and structure refinement details are summarized in Table 2 ▸.

## Supplementary Material

Crystal structure: contains datablock(s) I. DOI: 10.1107/S2414314620013103/hb4366sup1.cif

Structure factors: contains datablock(s) I. DOI: 10.1107/S2414314620013103/hb4366Isup2.hkl

Supporting information file. DOI: 10.1107/S2414314620013103/hb4366Isup3.cml

CCDC reference: 2032776

Additional supporting information:  crystallographic information; 3D view; checkCIF report

## Figures and Tables

**Table 1:** Hydrogen-bond geometry (Å, °)

*D*—H⋯*A*	*D*—H	H⋯*A*	*D*⋯*A*	*D*—H⋯*A*
N2—H2⋯N3^i^	0.89 (2)	2.11 (2)	2.9203 (13)	151 (1)
N3—H3*B*⋯O1^ii^	0.92 (2)	2.09 (1)	2.9516 (11)	157 (1)

Symmetry codes: (i) 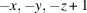 ; (ii) 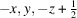 .

**Table 2:** Experimental details

Crystal data	
Chemical formula	C_9_H_13_N_3_O
*M*_r_	179.22
Crystal system, space group	Monoclinic, *C*2/*c*
Temperature (K)	100
*a*, *b*, *c* (Å)	24.7018 (6), 6.3093 (1), 13.2103 (3)
β (°)	118.0496 (8)
*V* (Å^3^)	1817.01 (7)
*Z*	8
Radiation type	Cu *K*α
μ (mm^−1^)	0.72
Crystal size (mm)	0.25 × 0.24 × 0.23

Data collection	
Diffractometer	Bruker APEXII CCD
Absorption correction	Multi-scan (*AXScale*; Bruker, 2016 ▸)
*T*_min_, *T*_max_	0.684, 0.754
No. of measured, independent and observed [*I* > 2σ(*I*)] reflections	15813, 1786, 1770
*R*_int_	0.019
(sin θ/λ)_max_ (Å^−1^)	0.618

Refinement	
*R*[*F* ^2^ > 2σ(*F* ^2^)], *wR*(*F* ^2^), *S*	0.036, 0.098, 1.07
No. of reflections	1786
No. of parameters	130
H-atom treatment	H atoms treated by a mixture of independent and constrained refinement
Δρ_max_, Δρ_min_ (e Å^−3^)	0.22, −0.22

Computer programs: *APEX3* and *SAINT* (Bruker, 2016 ▸), *SHELXS* (Sheldrick, 2008 ▸), *SHELXL* (Sheldrick, 2015 ▸), *OLEX2* (Dolomanov *et al.*, 2009 ▸), *CrystalExplorer17.5* (Mackenzie *et al.*, 2017 ▸), *Mercury* (Macrae *et al.*, 2020 ▸), and *publCIF* (Westrip, 2010 ▸).

## full crystallographic data

### Crystal data

**Table:**

C_9_H_13_N_3_O	*F*(000) = 768
*M**_r_* = 179.22	*D*_x_ = 1.310 Mg m^−^^3^
Monoclinic, *C*2/*c*	Cu *K*α radiation, λ = 1.54178 Å
*a* = 24.7018 (6) Å	Cell parameters from 9928 reflections
*b* = 6.3093 (1) Å	θ = 4.1–72.3°
*c* = 13.2103 (3) Å	µ = 0.72 mm^−^^1^
β = 118.0496 (8)°	*T* = 100 K
*V* = 1817.01 (7) Å^3^	Irregular, colourless
*Z* = 8	0.25 × 0.24 × 0.23 mm

### Data collection

**Table:**

Bruker APEXII CCD diffractometer	1786 independent reflections
Radiation source: Incoatec IMuS microfocus Cu tube	1770 reflections with *I* > 2σ(*I*)
Multi-layer optics monochromator	*R*_int_ = 0.019
φ and ω scans	θ_max_ = 72.4°, θ_min_ = 4.1°
Absorption correction: multi-scan (*AXScale*; Bruker, 2016)	*h* = −29→26
*T*_min_ = 0.684, *T*_max_ = 0.754	*k* = −7→7
15813 measured reflections	*l* = −16→16

### Refinement

**Table:**

Refinement on *F*^2^	Secondary atom site location: difference Fourier map
Least-squares matrix: full	Hydrogen site location: mixed
*R*[*F*^2^ > 2σ(*F*^2^)] = 0.036	H atoms treated by a mixture of independent and constrained refinement
*wR*(*F*^2^) = 0.098	*w* = 1/[σ^2^(*F*_o_^2^) + (0.0541*P*)^2^ + 1.3587*P*] where *P* = (*F*_o_^2^ + 2*F*_c_^2^)/3
*S* = 1.07	(Δ/σ)_max_ < 0.001
1786 reflections	Δρ_max_ = 0.22 e Å^−^^3^
130 parameters	Δρ_min_ = −0.22 e Å^−^^3^
0 restraints	Extinction correction: SHELXL2017/1 (Sheldrick 2015), Fc^*^=kFc[1+0.001xFc^2^λ^3^/sin(2θ)]^-1/4^
Primary atom site location: structure-invariant direct methods	Extinction coefficient: 0.0036 (3)

### Special details

**Table:**

Geometry. All esds (except the esd in the dihedral angle between two l.s. planes) are estimated using the full covariance matrix. The cell esds are taken into account individually in the estimation of esds in distances, angles and torsion angles; correlations between esds in cell parameters are only used when they are defined by crystal symmetry. An approximate (isotropic) treatment of cell esds is used for estimating esds involving l.s. planes.
Refinement. The hydrazide H2, H3A, and H3B atoms were located in difference-Fourier maps while all other hydrogen atoms were initially placed in calculated positions with their coordinates constrained to ride on their carrier atoms [C—H(aromatic) = 0.95?Å, C—H(methyl) = 0.98?Å]. The constraint Uiso(H) = 1.2Ueq(carrier) or 1.5Ueq(methyl carrier) was applied in all cases.

### Fractional atomic coordinates and isotropic or equivalent isotropic displacement parameters (Å^2^)

**Table:**

	*x*	*y*	*z*	*U*_iso_*/*U*_eq_	
O1	0.07812 (3)	0.16597 (12)	0.32634 (6)	0.0191 (2)	
N2	0.03227 (4)	0.11655 (14)	0.43722 (7)	0.0155 (2)	
H2	0.0261 (6)	0.151 (2)	0.4964 (12)	0.023*	
N3	0.00327 (4)	−0.07477 (14)	0.38183 (7)	0.0155 (2)	
H3A	0.0319 (7)	−0.162 (2)	0.3826 (12)	0.023*	
H3B	−0.0235 (6)	−0.037 (2)	0.3076 (13)	0.023*	
N1	0.18614 (4)	0.97723 (15)	0.64989 (8)	0.0218 (3)	
C5	0.08706 (4)	0.50333 (16)	0.56022 (8)	0.0147 (2)	
H5	0.060886	0.428200	0.582134	0.018*	
C6	0.11601 (4)	0.68522 (16)	0.61942 (8)	0.0155 (2)	
H6	0.109206	0.733294	0.680648	0.019*	
C1	0.15565 (4)	0.80037 (16)	0.58975 (8)	0.0156 (2)	
C2	0.16280 (5)	0.72556 (17)	0.49598 (9)	0.0174 (3)	
H2A	0.188258	0.801208	0.472528	0.021*	
C4	0.09528 (4)	0.42708 (16)	0.46894 (8)	0.0144 (2)	
C3	0.13326 (5)	0.54379 (17)	0.43786 (9)	0.0166 (2)	
H3	0.138925	0.496951	0.375132	0.020*	
C7	0.06791 (4)	0.22760 (16)	0.40464 (8)	0.0141 (2)	
C8	0.17404 (5)	1.06515 (18)	0.73873 (9)	0.0210 (3)	
H8A	0.131656	1.116555	0.704356	0.032*	
H8B	0.202237	1.183084	0.776385	0.032*	
H8C	0.180052	0.955197	0.795469	0.032*	
C9	0.22406 (5)	1.09916 (18)	0.61407 (10)	0.0222 (3)	
H9A	0.258398	1.011548	0.620994	0.033*	
H9B	0.239960	1.224711	0.662961	0.033*	
H9C	0.199403	1.143461	0.534079	0.033*	

### Atomic displacement parameters (Å^2^)

**Table:**

	*U*^11^	*U*^22^	*U*^33^	*U*^12^	*U*^13^	*U*^23^
O1	0.0220 (4)	0.0209 (4)	0.0193 (4)	−0.0049 (3)	0.0137 (3)	−0.0052 (3)
N2	0.0196 (4)	0.0142 (4)	0.0150 (4)	−0.0038 (3)	0.0101 (4)	−0.0026 (3)
N3	0.0172 (4)	0.0139 (4)	0.0148 (4)	−0.0023 (3)	0.0071 (4)	−0.0015 (3)
N1	0.0232 (5)	0.0217 (5)	0.0240 (5)	−0.0086 (4)	0.0140 (4)	−0.0071 (4)
C5	0.0137 (5)	0.0157 (5)	0.0156 (5)	0.0009 (4)	0.0077 (4)	0.0024 (4)
C6	0.0157 (5)	0.0171 (5)	0.0143 (5)	0.0016 (4)	0.0075 (4)	0.0006 (4)
C1	0.0130 (5)	0.0153 (5)	0.0160 (5)	0.0007 (4)	0.0049 (4)	0.0007 (4)
C2	0.0167 (5)	0.0182 (5)	0.0194 (5)	−0.0023 (4)	0.0103 (4)	0.0012 (4)
C4	0.0135 (5)	0.0143 (5)	0.0145 (5)	0.0010 (4)	0.0058 (4)	0.0012 (4)
C3	0.0176 (5)	0.0188 (5)	0.0165 (5)	0.0006 (4)	0.0105 (4)	−0.0001 (4)
C7	0.0125 (5)	0.0159 (5)	0.0133 (5)	0.0018 (4)	0.0055 (4)	0.0016 (4)
C8	0.0214 (5)	0.0185 (5)	0.0225 (5)	−0.0020 (4)	0.0098 (4)	−0.0054 (4)
C9	0.0197 (5)	0.0199 (6)	0.0270 (6)	−0.0059 (4)	0.0109 (5)	−0.0024 (4)

### Geometric parameters (Å, º)

**Table:**

O1—C7	1.2371 (12)	N2—H2	0.891 (15)
N2—N3	1.4187 (12)	N3—H3A	0.892 (17)
N2—C7	1.3444 (13)	N3—H3B	0.920 (15)
N1—C1	1.3715 (14)	C2—H2A	0.95
N1—C8	1.4506 (14)	C3—H3	0.95
N1—C9	1.4526 (14)	C5—H5	0.95
C5—C6	1.3835 (14)	C6—H6	0.95
C5—C4	1.3989 (14)	C8—H8A	0.98
C6—C1	1.4146 (14)	C8—H8B	0.98
C1—C2	1.4120 (14)	C8—H8C	0.98
C2—C3	1.3821 (15)	C9—H9A	0.98
C4—C3	1.3976 (14)	C9—H9B	0.98
C4—C7	1.4909 (14)	C9—H9C	0.98
			
C7—N2—N3	121.75 (8)	H3A—N3—H3B	109.9 (13)
C1—N1—C8	120.93 (9)	C1—C2—H2A	120
C1—N1—C9	120.31 (9)	C3—C2—H2A	120
C8—N1—C9	118.09 (9)	C2—C3—H3	119
C6—C5—C4	121.76 (9)	C4—C3—H3	119
C5—C6—C1	120.70 (9)	C4—C5—H5	119
N1—C1—C6	121.36 (9)	C6—C5—H5	119
N1—C1—C2	121.27 (9)	C1—C6—H6	120
C2—C1—C6	117.37 (9)	C5—C6—H6	120
C3—C2—C1	120.90 (9)	N1—C8—H8A	109
C5—C4—C7	124.79 (9)	N1—C8—H8B	109
C3—C4—C5	117.50 (9)	N1—C8—H8C	109
C3—C4—C7	117.69 (9)	H8A—C8—H8B	109
C2—C3—C4	121.73 (9)	H8A—C8—H8C	109
O1—C7—N2	121.71 (10)	H8B—C8—H8C	109
O1—C7—C4	121.71 (9)	N1—C9—H9A	109
N2—C7—C4	116.58 (9)	N1—C9—H9B	109
N3—N2—H2	114.0 (9)	N1—C9—H9C	109
C7—N2—H2	124.2 (9)	H9A—C9—H9B	109
N2—N3—H3A	108.3 (10)	H9A—C9—H9C	109
N2—N3—H3B	105.4 (8)	H9B—C9—H9C	109
			
C8—N1—C1—C2	173.90 (10)	C1—N1—C9—H9A	−64
C8—N1—C1—C6	−6.35 (15)	C1—N1—C9—H9B	176
C9—N1—C1—C2	3.50 (16)	C1—N1—C9—H9C	56
C9—N1—C1—C6	−176.75 (10)	C8—N1—C9—H9A	125
N3—N2—C7—O1	−1.50 (15)	C8—N1—C9—H9B	5
N3—N2—C7—C4	179.43 (9)	C8—N1—C9—H9C	−115
N1—C1—C2—C3	178.16 (11)	C7—N2—N3—H3A	54.0 (10)
C6—C1—C2—C3	−1.60 (16)	C7—N2—N3—H3B	−63.6 (11)
N1—C1—C6—C5	−177.96 (10)	H2—N2—N3—H3A	−123.2 (14)
C2—C1—C6—C5	1.79 (15)	H2—N2—N3—H3B	119.3 (15)
C1—C2—C3—C4	0.07 (18)	H2—N2—C7—O1	175.4 (11)
C2—C3—C4—C5	1.28 (16)	H2—N2—C7—C4	−3.7 (11)
C2—C3—C4—C7	−177.07 (10)	N1—C1—C2—H2A	−2
C3—C4—C5—C6	−1.08 (15)	C6—C1—C2—H2A	178
C7—C4—C5—C6	177.14 (10)	N1—C1—C6—H6	2
C3—C4—C7—O1	−0.02 (15)	C2—C1—C6—H6	−178
C3—C4—C7—N2	179.06 (10)	C1—C2—C3—H3	−180
C5—C4—C7—O1	−178.23 (10)	H2A—C2—C3—C4	−180
C5—C4—C7—N2	0.84 (15)	H2A—C2—C3—H3	0
C4—C5—C6—C1	−0.47 (16)	H3—C3—C4—C5	−179
C1—N1—C8—H8A	−65	H3—C3—C4—C7	3
C1—N1—C8—H8B	175	C3—C4—C5—H5	179
C1—N1—C8—H8C	55	C7—C4—C5—H5	−3
C9—N1—C8—H8A	106	C4—C5—C6—H6	180
C9—N1—C8—H8B	−14	H5—C5—C6—C1	180
C9—N1—C8—H8C	−134	H5—C5—C6—H6	0

### Hydrogen-bond geometry (Å, º)

**Table:**

*D*—H···*A*	*D*—H	H···*A*	*D*···*A*	*D*—H···*A*
N2—H2···N3^i^	0.89 (2)	2.11 (2)	2.9203 (13)	151 (1)
N3—H3*B*···O1^ii^	0.92 (2)	2.09 (1)	2.9516 (11)	157 (1)

Symmetry codes: (i) −*x*, −*y*, −*z*+1; (ii) −*x*, *y*, −*z*+1/2.
